# Alteration of Fatty Acid Oxidation in Tubular Epithelial Cells: From Acute Kidney Injury to Renal Fibrogenesis

**DOI:** 10.3389/fmed.2015.00052

**Published:** 2015-08-05

**Authors:** Noémie Simon, Alexandre Hertig

**Affiliations:** ^1^IMSERM UMR_S1155, Rare and Common Kidney Diseases, Remodeling and Tissue Repair, Hôpital Tenon, Paris, France; ^2^UMR S 1155, UPMC Sorbonne Université Paris 06, Paris, France

**Keywords:** fatty acid oxidation, epithelium, fibroblasts, acute kidney injury, chronic kidney diseases, fibrosis

## Abstract

Renal proximal tubular cells are the most energy-demanding cells in the body. The ATP that they use is mostly produced in their mitochondrial and peroxisomal compartments, by the oxidation of fatty acids. When those cells are placed under a biological stress, such as a transient hypoxia, fatty acid oxidation (FAO) is shut down for a period of time that outlasts injury, and carbohydrate oxidation does not take over. Facing those metabolic constraints, surviving tubular epithelial cells exhibit a phenotypic switch that includes cytoskeletal rearrangement and production of extracellular matrix proteins, most probably contributing to acute kidney injury-induced renal fibrogenesis, thence to the development of chronic kidney disease. Here, we review experimental evidence that dysregulation of FAO profoundly affects the fate of tubular epithelial cells, by promoting epithelial-to-mesenchymal transition, inflammation, and eventually interstitial fibrosis. Restoring physiological production of energy is undoubtedly a possible therapeutic approach to unlock the mesenchymal reprograming of tubular epithelial cells in the kidney. In this respect, the benefit of the use of fibrates is uncertain, but new drugs that could specifically target this metabolic pathway, and, hopefully, attenuate renal fibrosis merit future research.

## Introduction

Renal fibrosis is the final common pathway to all chronic kidney diseases (CKD), suggesting that a biological switch is activated in any context of injury, suppressing noble renal functions long-term and reactivating embryonic-like mesenchymal ones (1). Such a switch would obviously be a major source of new therapeutics.

Although the consequence of renal fibrosis is the loss of epithelial functions, it is defined morphologically in organs by an expansion of the connective tissue (swelling of interstitial myofibroblasts and excess of deposition of extracellular matrix). At the tubular cell scale, it consists of an atrophic type lesion with a thickening of basement membranes and a flattened epithelium, suggesting dedifferentiation. However, evidence has accumulated to show that proximal tubular epithelial cells (PTC) are not uninvolved in the process of interstitial fibrogenesis: placed under biological stress, they can undergo phenotypic changes, acquire active mesenchymal functions, and even proliferate (2), and hence, contributing to the synthesis of extracellular matrix proteins and perpetuating fibrosis (3, 4). As epithelial cells have two equilibrium states (epithelial during homeostasis, mesenchymal in pathological situations), some authors often refer to this bi-stability as epithelial-to-mesenchymal transition (EMT) (5), analogous with the profound phenotypic changes observed in primary epiblasts during embryogenesis (or at the edge of a carcinoma). However, the term is confusing, since in embryos and tumors, EMT is essential to disperse cells. Whether EMT becomes so intense in adult fibrotic kidneys that epithelial cells leave their tubular structure and provide *de novo* myofibroblasts has been vigorously debated (6, 7). Over the last decade, experimental research has shown that pericytes, not epithelial cells, are considered the most significant source of such *de novo* myofibroblasts (8), and the contribution of EMT in organ fibrogenesis is seen as local rather than diffuse, i.e., relevant within tubular structures (9). However, the process in humans is unknown, and EMT-like biological changes might significantly contribute, even at the local level.

Hypoxia is one of the many biological stresses that may tip the balance toward a mesenchymal program. Segment 3 renal PTC (the *pars recta*, located at the corticomedullary junction) in particular are very hypoxia sensitive. This is because of a combination of the locally lowest oxygen pressure in this anatomic segment on the one hand (10, 11), with a high-energy consumption linked to highly specialized ATP-consuming transporters on the other (12, 13). Like most of the highly metabolic cells, the preferred energy fuel is the one with the highest ATP production: fatty acid oxidation (FAO) (14). The aim of this review is to provide an insight into how down-regulation of FAO observed during acute kidney injury (AKI) – not only during ischemic AKI but also in other experimental conditions – precedes EMT and constitutes a major alteration of cell metabolism, which drives the mesenchymal transition. Drugs promoting or restoring FAO are therefore promising.

## AKI as a Trigger of Renal Fibrogenesis: A Shift in the Paradigm

Recently, the concept of AKI has been redefined. This term now encompasses a range of renal impairment, from even small changes in function (serum creatinine or urinary output) to the necessity for renal replacement therapy. AKI can be secondary to a large spectrum of causes. Histological features of AKI often include lesions of acute tubular necrosis (ATN), a renal lesion that was long thought to be fully reversible. However, this paradigm shifted some years ago toward the notion that the repair of ATN could be “maladaptive” and initiate fibrogenesis at a molecular level, even when morphology had (at least transiently) returned to normal (15). Similarly, increasing evidence in kidney transplantation suggests that ischemic episodes are connected to transplant fibrosis (16). Mechanisms at stake in the interconnection between AKI and CKD are an intense area of research. At present, three major abnormalities have been found to be associated with the fibrotic outcome of a transient AKI: (a) the epigenetic silencing of *RASAL1*, a proliferation inhibitor, in myofibroblasts; (b) the cell cycle arrest in G2/M in tubular epithelial cells (the G2/M phase is where the epithelial cell function is closer to a mesenchymal one); and (c) down-regulation of FAO in tubular epithelial cells (14, 17, 18). These mechanisms are not exclusive of each other.

## Fatty Acid Oxidation: A Brief Description

A fatty acid (FA) is a carboxylic acid with a long aliphatic (as opposed to aromatic) tail. It can be produced by FA synthesis or by hydrolysis of triglycerides or phospholipids; conversely, a triglyceride is a storage (triester) form of FA. Triglyceride overload leads to lipid droplet formation. Historical studies by Weidemann and Krebs were the first to report that in dog kidneys these droplets can be used in case of scarcity. Thus, FAO may help PTC to adapt to energy demand (19).

**Figure 1:**
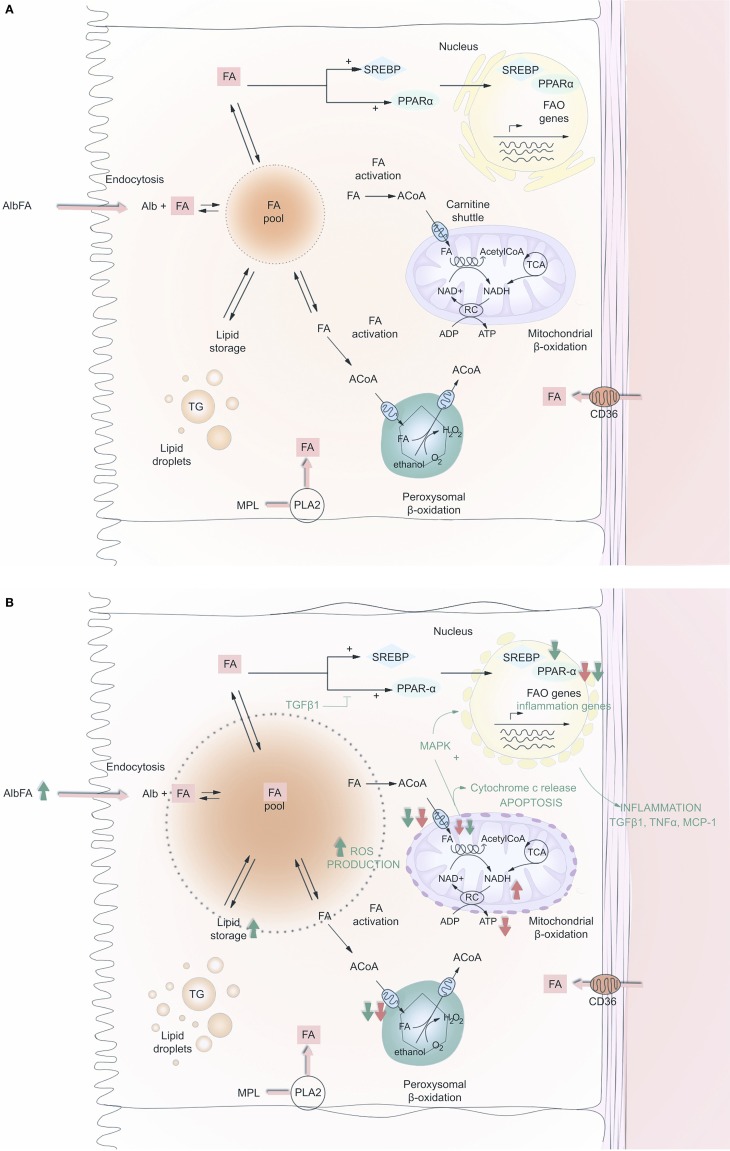
**Fatty acid (FA) metabolism in renal PTC before (A) and after (B) acute kidney injury**. FA may enter the cell either at the apical or at the baso-lateral side, free or albumin bound. They may also be produced after hydrolysis of membrane phospholipids, by phospholipase A2. Intracellular FA is then routed to anabolic or catabolic pathways; FA is stored in the global triglyceride pool or oxidized in mitochondria or peroxisome to produce ATP. The carnitine shuttle gives access to the matrix of these two organelles. FAO enzymes are positively retro-controlled by FA accumulation at the transcriptional level by the activation of SREBP1c and PPAR-α. On **(B)**, red and green arrows indicate what is being down-regulated (down arrows) or up-regulated (up arrows) during AKI and fibrosis, respectively. Abbreviations: FA, fatty acid; CoA, CoenzymeA; ACoA, acyl-CoenzymeA; Alb, albumin; SREBP, sterol regulatory element-binding protein-1c; PPAR-α, peroxisome proliferator activated receptor-alpha; MAPK, mitogen-activated protein kinase; FAO, fatty acid oxidation; MPL, membrane phospholipid; ROS, reactive oxygen species; NAD, nicotine adenosine dinucleotide; RC, respiratory chain; PLA2, phospholipase A2; TG, triglyceride; TGF-β1, transforming growth factor β1; ADP, adenosine diphosphate; ATP, adenosine triphosphate; TNF α, tumor necrosis factor α; MCP-1, monocyte chemo-attractant protein-1.

Fatty acid oxidation occurs in the mitochondrial and peroxisomal compartments. FAs must first be supplied, either by extracellular uptake through the FA transport protein CD36 (20), or by *in situ* cytosolic synthesis, or by the deacylation of cellular phospholipids under the action of phospholipase A2 (PLA2). Second, FAs has to be transported from the cytosol to the respective organelles in order to be oxidized and provide the cell with ATP (Figure 1). The outer membranes of the mitochondrion and the peroxisome are not permeable to long-chain FA, so FAs need to use a specific transporter called the carnitine shuttle. For this to occur, they need to be “activated” by coenzyme A in the cytosol, under the action of an acyl-CoA synthetase, which is located on the outer membrane of the organelle. The resulting long-chain acyl-CoA products will then interact with a carnitine molecule to regenerate coenzyme A and produce a long-chain acyl carnitine (LCAC), to which the outer membrane is readily permeable. This step also requires the rate-limiting enzyme of the carnitine shuttle, the carnitine palmitoyl-transferase 1 (CPT-1), similarly located on the outer membrane. LCAC is eventually able to cross the inner (impermeable) membrane thanks to the carnitine-acyl-carnitine translocase. The carnitine palmitoyl-transferase 2 then ensures a reverse reaction regenerating the carnitine molecule using coenzyme A, resulting again in an acyl-CoA product, which will undergo β-oxidation in the peroxisome and the mitochondrion. The system is complex but still economic in that the carnitine molecule will be transported back to the cytoplasm by the same shuttle. Oxidation (the loss of an electron) then occurs because electron carriers flavine adenine dinucleotide (FAD) and nicotine adenosine dinucleotide (NAD) will accept an electron from acyl-CoA, and hence, be reduced to FADH and NADH, respectively. Since these reactions occur close to the inner membrane, where the electron transfer chain is located, FAD and NAD are instantly regenerated. The term β-oxidation refers to the position of the carbon group being oxidized. The energy yield of FA β-oxidation is very high, with an average of 106 ATP equivalents per FA, as opposed to 36 during the oxidation of carbohydrates.

## Energy Metabolism in Renal Proximal Tubular Cells during AKI

Several studies have reported on a triglyceride overload following endotoxic, toxic, and ischemic kidney injury (21, 22). FA accumulation is observed after ischemic AKI (23), most probably because of a lack of oxidative substrates to accomplish FAO (24). In the mitochondrion, the rate-­controlling step of the FAO is the oxidative reaction catalyzed by 3-hydroxyacyl-CoA dehydrogenase. As explained above, and in Figure 1A, this enzyme requires NAD as oxidant under its oxidized form (NAD^+^), which is regenerated by the respiratory chain located in the inner mitochondrial membrane in normoxic conditions, as oxygen acts as the terminal electron acceptor. Likewise, acyl-CoA oxidase, the key enzyme for β-oxidation in the peroxisome, uses oxygen as a substrate to perform FAO. Therefore, hypoxia dramatically decreases the NAD^+^/NADH ratio. Short of fundamental substrates for the critical steps of FAO, FAO also dramatically decreases. Reperfusion should theoretically lead to the restoration of a functional FAO in the aftermath of an acute cellular stress. However, Gulati et al. demonstrated that in the peroxisome compartment, FAO enzymes were not only inhibited during the ischemic phase but also during reperfusion, because of a proteolytic degradation process, particularly affecting the acyl-CoA oxidase (25). In mitochondria, ischemia/reperfusion injury (IRI) also leads to a decline in the activity of CPT-1, the rate-limiting enzyme of the carnitine shuttle; therefore, cutting off FA supplies in the mitochondrial matrix (26). Feldkamp et al. also observed that PTC isolated from rabbit kidneys exhibit a free FA accumulation during hypoxia and reperfusion, and an ATP decrease during hypoxia that outlasts re-oxygenation (27). Although it is reasonable to think that in the absence of its usual fuel (FA), PTC would turn to glucose oxidation to produce ATP even at a lower rate, and resume even minimal epithelial functions, the sustained alteration of FAO is not followed by such a take over in the context of AKI, either *in vitro* or *in vivo* (14). With prolonged ATP shortage, proliferation and mesenchymal reprograming of epithelial cells could thus be an energy efficient route to survival. Resetting the tools for ATP production, and in particular for FAO, is thus a promising approach. Of note, whether this metabolic pathway is generally necessary for pericytes, or more particularly enables them to maintain a stable phenotype instead of becoming fibroblasts, is not known.

## Resetting Fatty Acid Oxidation after AKI

**Figure 2:**
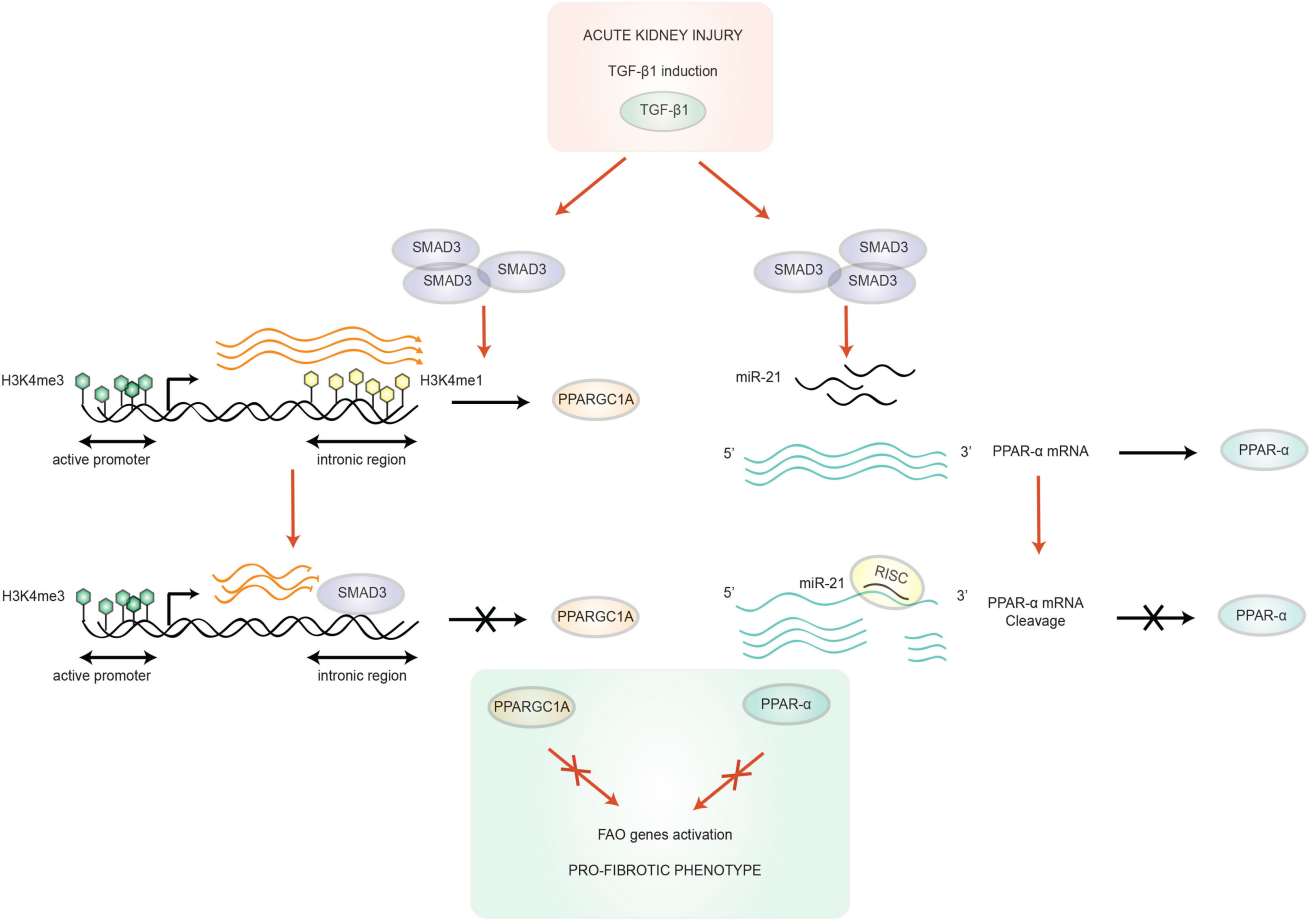
**Transforming growth factor-**β**1/SMAD3 mediated-regulation of FAO**. TGF-β1 is up-regulated after acute kidney injury, which activates SMAD3, which in turn can bind to an intronic region of the PPARGC1A gene. SMAD3 binding overlaps with the active enhancer histone tail modification H3K4me1 of this sequence, resulting in the blocking of the progression transcription machinery. In addition, this region is also annotated as an active enhancer in human kidney PTC (14). SMAD3 can also target PPAR-α, the other key regulator gene of FAO, through microRNA (miR-21) overexpression. miR-21 silences PPAR-α by recognition of an octamer sequence complementary to miR-21 seed region in the 3′UTR of PPAR-α mRNA (32). These two mechanisms cooperate in the acquisition of a pro-fibrotic phenotype. Abbreviations: PPAR-α, peroxisome proliferator activated receptor-alpha; PPARGC1A, PPAR-γ co-activator-1a; RISC, RNA-induced silencing complex; TGF-β1, transforming growth factor β1.

Peroxisome proliferator activated receptor-alpha (PPAR-α) is a transcription factor predominantly expressed in metabolically very active tissues, such as renal PTC, and has been shown to control FAO. In homeostasis, endogenous levels of FA act directly on PPAR-α as natural activators of this ligand-activated receptor superfamily member. PPAR-α increases the transcription of genes encoding FAO enzymes, and also acts upstream by stimulating cellular FA uptake through the modulation of the FA translocase CD36 (28). Conversely, during AKI, PPAR-α mRNA, and its DNA binding activity were found to decrease, as was the availability of its tissue specific co-activator PPAR-γ co-activator-1a (PPARGC1A) (29–31). Kang et al. have reported that transforming growth factor β1 (TGF-β1), a major player in kidney fibrosis, and a master inducer of EMT, can inhibit PPAR-α and PPARGC1A, key transcription factors of FAO genes. It logically results in a down-regulation of CPT-1 and triglyceride overload. How TGF-β1 suppresses PPAR-α, and PPARGC1A seems to be epigenetically regulated. MicroRNA-21 (miR-21), a downstream target of Smad3 (32), is able to silence PPAR-α (33). Strikingly, anti-miR-21 failed to suppress renal fibrosis in PPAR-α^−/−^ mice, incidentally underlining the major role of PPAR-α/FAO in the process of renal fibrogenesis. In addition, chromatin immunoprecipitation (CHiP) studies revealed that Smad3 can bind to an intronic area of the PPARGC1A promoter, at a position where the DNA is enriched in the histone mark H3K4me1 (read: mono-methylation of lysine 4 in histone 3, a mark usually associated with activation of transcription). By overlapping with this region, Smad3 could thus impede epigenetic activation of PPARGC1A (14) (Figure 2).

## Fatty Acid Oxidation and Epithelial-to-Mesenchymal Transition

A genomic study of hepatocellular carcinomas showed that down-regulation of critical genes involved in FAO (hydroxyl-acyl-CoA dehydrogenase and acyl-coA oxidase) correlates to a “dedifferentiation” state of tumoral tissue, which also involves up-regulation of *SNAIL*, a major regulator of EMT (34). More recently, a study revealed in a human renal cell line (HK2) that lipid accumulation and FAO decrease precede glucose-induced morphological changes by 48 h, in addition to the cytoskeletal switch typical of EMT [loss of E-cadherin and acquisition of alpha-smooth muscle actin (α-SMA)]. Although this does not prove a causal relationship, it is interesting to note that the silencing of acetyl-coA carboxylase 2 (ACC2), the enzyme catalyzing the carboxylation of acetyl-coA into malonyl-coA, a potent inhibitor of CPT-1, abrogated glucose-induced morphological changes, cytoskeletal switch, and increased the rate of FA (35). Kang et al. showed that a global down-regulation of genes involved in FA metabolism was also found *ex vivo* in human fibrotic kidneys compared to controls. They also reported that PTC treated by the CPT-1 inhibitor Etomoxir undergo morphologic and genomic changes, with the expression of more mesenchymal genes, such as *ACTA2* (encoding α-SMA), *VIM* encoding vimentin, an intermediate filament, and *COL1A1* and *COL3A1* encoding fibrillary collagens (14). Thus, accumulation of lipids and/or decreased FAO could participate in the mesenchymal reprograming of epithelial cells in the kidney, a non-lipogenic tissue. We will now briefly discuss why the former, taken alone, is not considered a sufficient trigger of EMT.

## Renal Fibrosis, Fatty Acids, and Inflammation

“Lipotoxicity” is defined by FA accumulation on non-adipose tissues. Lipid accumulation reflects an imbalance between FA utilization and FA supplies, as in the case of FAO inhibition, and because of triglyceride buffer FA excess, overload is often visible as lipid droplets. In human diseases as well as in animal models, lipid deposition has repeatedly been observed in PTC (36) (in glomerulonephritis models, for example, glomerular injury allows PTC to reabsorb albumin/FA complexes, eventually resulting in cell FA accumulation).

Fatty acid accumulation could play a role in the systemic manifestations of “metabolic” pathologies, such as diabetes mellitus and obesity, where free or albumin-bound FA blood levels are increased (37), CD36 is overexpressed due to glucose exposure, and FA synthesis also increases [in OVE26 and Akita type 1 diabetic mice, animals exhibit an increase in sterol regulatory element-binding protein-1c (SREBP-1c) mRNA, a protein known to up-regulate FA synthase and acetyl-coA carboxylase]. This does not necessarily increase FAO; however, for example, acetyl-coA carboxylase inhibits FAO, and malonyl-coA inhibits CPT-1, so overall excess in FA might eventually inhibit FAO (38, 39). This hypothesis is also supported by protection of SREBP1c KO from high-fat diet-associated tubulo-interstitial injuries (38).

In addition, excess of FA may impact epithelial cells independently from the FAO pathway. Albumin-bound FAs have been reported to activate PPAR-δ, dose-dependently, and alter mitochondrial function, leading to cytochrome *c* release and caspase 3 activation (40, 41), and *in vivo*, to enhance tubular inflammation via a pro-inflammatory metabolite (42). Soumura et al. also showed that palmitic acid treatment induces an up-­regulation of the expression of monocyte chemo-attractant protein-1 (MCP-1) and leads to activation of two pro-inflammatory pathways thanks to phosphorylation of MAPKs (ERK, p38, and JNK) and IκB, thus promoting the nuclear translocation of NF-κB (43). Katsoulieris et al. not only corroborated these results but also demonstrated that palmitic acid overload in a renal PTC model induces endoplasmic reticulum (ER) stress (44). ER stress, activated by unfolded or misfolded proteins or protein trafficking, is a well-known phenomenon in the development and progression of kidney disease. In this case, palmitic acid can cause ER stress by H_2_O_2_ production and C/EBP homologous protein (CHOP) expression (44). Oxidative and ER stress, and apoptosis unite to create a pro-inflammatory state in the vicinity of renal PTC (45). Last, knocking-out the scavenger receptor CD36 results in a reduction of activated NF-κB and oxidative stress level (46) in mice subjected to unilateral ureteral obstruction and a high-fat diet. Overall, lipotoxicity probably exists and contributes to epithelial injury, either directly through the activation of an apoptotic signal or indirectly by promoting the influx of inflammatory cells, a major factor in fibrosis.

Nevertheless, there is some doubt regarding the importance of lipid overload *per se*. Although cell-specific overexpression of CD36 in tubular epithelial cells from mice leads to lipid accumulation by the age of 8 weeks, it is not sufficient to drive spontaneous renal fibrogenesis, and more importantly, it does not enhance the susceptibility to renal fibrosis in two different animal models (diabetic nephropathy and folic acid nephropathy) (14). Within epithelial cells, it is thus thought that peroxisomal/mitochondrial defects in energy production are more detrimental than the lipid accumulation in the cytoplasm.

## PPAR-α as New Therapeutic Target

Proximal tubular epithelial cell-specific PPAR-α overexpression in mice was found to be sufficient to maintain FAO and conferred protection against IRI (47). Agonists of PPAR-α have been proposed for therapeutic use to prevent cisplatin-induced AKI, free FA accumulation, and ischemia–reperfusion injury (30, 48, 49). The first class tested was fibrates, with mixed results. Bezafibrate displays a protective effect against apoptosis in a cellular model and attenuates intracellular free FA accumulation (48). Takahashi et al. showed in a high-fat diet model that pre-treatment with clofibrate at a low dose (but not at a high dose) protects against free FA toxicity (30). PPAR-α agonists administered 5 days before injury in a rat model of renal IRI were found to regulate acyl-coA oxidase at the transcriptional and protein levels and to attenuate tubular necrosis. In a bilateral ischemia rat model, it has also been reported that PPAR-α agonists, clofibrate, fenofibrate, and WY14643, reduce renal dysfunction and inflammation related to IRI (49, 50). Of note, in the case of fenofibrate, protective effect was lost in PPAR-α^−/−^ (49, 51). The effect of these reno-protective mechanisms was partially elucidated by Tanaka and co-workers, who demonstrated in a high-fat diet mice model that fenofibrate not only enhances lipolysis by overexpressing CPT-1, acyl-coA oxidase, and medium-chain acyl-CoA dehydrogenase but also inhibits the expression of the pro-fibrotic factors plasminogen activator inhibitor-1 (PAI-1) and MCP-1 (52). In a hypertension rat model fed with a high-fat diet, fenofibrate treatment induced PPAR-α expression and decreased apoptosis (53). Despite these promising data, the efficacy of fibrates in the clinic is so far limited to the reduction of albuminuria in type 2 diabetic patients. However, this data should be interpreted with caution, since the primary endpoint of the study was not fulfilled. No additional benefit was demonstrated with Gemfibrozil in two different large cohort studies (54, 55). Importantly, however, a recent meta-analysis concluded that fibrates played a preventive role in cardiovascular events in CKD patients, and confirmed albuminuria reduction (56). The effects of fibrates on the progression rate of CKD are still unknown since no study has yet been designed for this primary outcome (57).

Another approach to increase FAO would be to facilitate the transport of FA into the mitochondrial matrix. A treatment combining carnitine and 5-aminoimidazole-4-carboxyamide ribonucleoside (AICAR), which indirectly activates CPT-1 through the adenosine monophosphate-activated protein kinase (AMPK), was found to improve renal function after IRI, suggesting that FAO can be artificially stimulated (58). Propionyl l-carnitine also seems to be a promising candidate in IRI lesion prevention, through refeeding the carnitine shuttle and indirectly the Krebs cycle (59).

## Conclusion

Fatty acid oxidation is unsurprisingly shut down during oxygen deprivation, a major cause of AKI. The observation that FAO does not properly resume after the injury suggests that tubular epithelial cells have to cope with a sustained lack of energy (Figure 1B). This could be one reason among others why cells switch from a highly demanding epithelial phenotype, to a more economical, mesenchymal one, and why even a transient AKI increases the risk of developing CKD. Lipid accumulation resulting from FAO stunning probably also contributes indirectly to enhanced fibrogenesis, by promoting inflammation. It is reasonable to assume that unlocking the state of metabolic sideration of PTC would permit the rescue of an epithelial phenotype, and reversion to pro-fibrotic mesenchymal functions. Playing a major role in the regulation of FAO, the PPAR-α/PPARGC1A ensemble is a reasonable therapeutic target for the future.

## Author Contributions

NS wrote the draft and designed the figures. AH suggested the plan of the review and finalized the manuscript.

## Conflict of Interest Statement

The authors declare that the research was conducted in the absence of any commercial or financial relationships that could be construed as a potential conflict of interest.
